# Cerebro-Spinal Meningitis

**Published:** 1870-05

**Authors:** Thos. C. Murphy

**Affiliations:** Grand Valley, Ill.


### ARTICLE IV.—*Cerebro-Spinal Meningitis.* By THOS. C. MURPHY, M. D., Grand Valley, Ill.

In the April number of the *Journal* is an article calling your attention to the importance of Cerebro-Spinal Meningitis. The Doctor asks you to lay before your readers *all* that is known on the subject. I should say the single word *all* covers a large field. How to distinguish Meningitis from typhoid is another question. Cerebro-Spinal Meningitis should never be mistaken for typhoid fever. The suddenness of the attack, intense headache, absence of febrile movement in the beginning of the attack, headache increasing as the disease advances, and the occurrence of symptoms denoting inflammation of the meninges of the brain and spinal cords, should distinguish it from typhoid fever. In typhoid the attack is gradual; we have diarrhœa, meteorism, iliac tenderness, and the peculiar expression of the countenance, which, once seen, cannot be forgotten. In Meningitis we have tonic contraction of the muscles of the neck and back, rachialgia, convulsions, and delirium, occurring not infrequently. In typhoid fever the rise of temperature is gradual, and reaches 104 degrees Fahrenheit on the fourth day. In Meningitis the onset is abrupt, and the temperature ranges from 100 to 101 degrees Fahrenheit, and never reaches as high as in typhoid fever. Nausea and vomiting are prominent symptoms in Meningitis. The clinical history of the two differ widely.

### CAUSES OF MENINGITIS.

During the late war, filth and crowd poisons seemed to be active agents in the production of this disease. Among the soldiers of the U. S. army, recruiting depots were more liable to attack than were troops in the field; showing that filth and crowd poisons are active agents in producing Meningitis as well as typhoid fever. But the poison of the one cannot produce the other; they are two distinct poisons, and always will be.

### PROGNOSIS.

Meningitis must be regarded as a grave disease. Of the number of persons attacked, the majority die; in fact, many cases are struck down at once, as in malignant scarlet fever. Such is the virulent nature of the poison that, at the onset, death steps in during the first few hours of the attack.

### MORBID ANATOMY.

(From the Report of the Surgeon General, U. S. Army.)

"There were two classes of cases brought under the observation of this department. In the first, the autopsy disclosed grave anatomical lesion of the cerebro-spinal axis, accumulations of serum, sero-pus, pus or tough yellow lymph, especially in the ventricles about the base of the brain, and in the upper part of the spinal canal. In the second class of cases, no perceptible anatomical lesion in the cerebro-spinal axis was observable. These two groups of cases rest upon equally reliable evidence, and are not to be disposed of on the supposition that the latter represents merely an early stage of the disease. Since, it is to be remarked that both anatomical conditions appear to have been found indifferently, in protracted cases as well as those which prove suddenly fatal." It has been well said of this disease, it is easier to say what it is not than what it is.

### TREATMENT.

There is no antidote known to the profession that will counteract the specific ætic poison, nor can it be expelled by elimination. The indications are to stay, if possible, the progress of the disorder, and sustain the vital powers. A hot bath, in which the patient is to remain a short time only, and to be immediately wrapped in blankets, often gives some relief. Hypodermic injections of Morphia are called for when the pain is intense. The bowels should be kept open by cathartics; nutritious food must be given, beef tea, milk, etc. It seems as if the medical dice box has been rattled in vain for a specific (so called) for this disease. One tries blisters and Belladonna, another tries Potass Bromide. The last seems to be a remedy of some use in this disease, at least it should have a fair trial. Cold to the head is of great value. In cases where there is great depression of the nervous system, the tincture of cantharides is of great value from its stimulant effects, and in such cases it is indicated. (I believe, to Prof. Allen, of Rush Medical College, is due the credit of reviving the use of the tincture of cantharides.) When there are so many agents recommended, I think the physician should use his own judgment, and treat each case according to the indications, and not be crying out for a sign. No sign shall be given ye. As for bringing before the profession all that is known on the subject of Meningitis, one medical journal is too small, and it would not be just the thing. Your correspondent (G. I. S.) can find all he seeks for in some of the practical works on Medicine; say Aitken's, Flint's, Bennett's. Much valuable information can be had from good medical journals. From the above works, a moderate share of knowledge may be drawn, but — the words of the Queen to Solomon — the half has not yet been told, (or some one else said so.)

Why spend valuable time in trying to find out the name of a disease? You cannot treat a disease according to its name or location, but according to what's the matter. Treat according to the indication all the time. Study the clinical history of the two diseases. Make it a point to have a little clinic of your own. Let each case be your hospital. Think; observe.

